# The societal costs of dementia in Sweden 2012 – relevance and methodological challenges in valuing informal care

**DOI:** 10.1186/s13195-016-0215-9

**Published:** 2016-11-18

**Authors:** Anders Wimo, Linus Jönsson, Laura Fratiglioni, Per Olof Sandman, Anders Gustavsson, Anders Sköldunger, Lennarth Johansson

**Affiliations:** 1 Aging Research Centre, Department of Neurobiology, Care Sciences and Society (NVS), Karolinska Institutet and Stockholm University, Stockholm, Sweden; 2 Division of Neurogeriatrics, Department of Neurobiology, Care Sciences and Society (NVS), Karolinska Institutet, Huddinge, Stockholm Sweden; 3 Centre for Research & Development, Uppsala University/County Council of Gävleborg, Gävle, Sweden; 4 Division of Caring Sciences, Department of Neurobiology, Care Sciences and Society (NVS), Karolinska Institutet, Stockholm, Sweden; 5 Department of Nursing, Umeå University, Umeå, Sweden; 6 Department of Health Sciences, Luleå University of Technology, Luleå, Sweden; 7 Stockholm Gerontology Research Centre, Stockholm, Sweden

**Keywords:** Costing study, Cost of illness, Dementia, Alzheimer’s disease, Sweden

## Abstract

**Background:**

In this study, we sought to estimate the societal cost of illness in dementia in Sweden in 2012 using different costing approaches to highlight methodological issues.

**Methods:**

We conducted a prevalence-based cost-of-illness study with a societal perspective.

**Results:**

The societal costs of dementia in Sweden in 2012 were SEK 62.9 billion (approximately €7.2 billion, approximately US$9.0 billion) or SEK 398,000 per person with dementia (approximately €45,000, approximately US$57,000). By far the most important cost item is the cost of institutional care: about 60% of the costs. In the sensitivity analysis, different quantification and costing approaches for informal care resulted in a great variation in the total societal cost, ranging from SEK 60 billion (€6.8 billion, US$8.6 billion) to SEK 124 billion (€14.1 billion, US$17.8 billion).

**Conclusions:**

The societal costs of dementia are very high. The cost per person with dementia has decreased somewhat, mainly because of de-institutionalisation. The majority of the costs occur in the social care sector, but the costing of informal care is crucial for the cost estimates.

## Background

Dementia disorders are chronic, long-lasting diseases that impair cognition, social capacity and daily functioning of the persons affected. As a consequence, dementia heavily influences the situation not only for the patients themselves but also for family members and other next of kin. Furthermore, dementia disorders are also very resource-demanding and costly, which, in a setting of scarce public resources and, in many countries, ongoing changes in care systems, puts great stress on decision makers and budget holders. The worldwide societal cost of dementia care was estimated to be US$604 billion for 36 million persons with dementia (PWD) in 2010 [1, 2]. The magnitude and allocation of resource use and costs are of interest when present and future care is discussed in terms of organisation, volume and financing.

The situation in dementia care is dynamic. Demographic prognoses predict a rapid increase in the number of people affected [3–5], making the situation even more urgent, and apocalyptic scenarios of the ‘dementia bomb’ and suchlike have been presented [6]. In contrast, some studies have indicated that age-specific incidence and/or prevalence of dementia and cognitive impairment might have decreased in high-income countries such as the United States, the United Kingdom, The Netherlands and Sweden [7–11]. Symptomatic drug treatment for Alzheimer’s disease has been available for almost 20 years. Studies on potential disease-modifying agents have so far not been successful, although there are still many compounds in clinical development [12, 13]. Because of changes in long-term care policies, as well as strategies and pressures for cost containment in health care and social care systems, there is a trend towards de-institutionalisation in some countries [14].

In 1991, the cost of illness (COI) in Sweden was estimated to be 31 billion Swedish krona (SEK) for a dementia population of 154,000 (which is probably an overestimate of the prevalence) [15]. For 2000, the cost was estimated to be SEK 38 billion for 133,000 PWD [16], and for 2005, it was estimated as SEK 50 billion for 142,000 PWD [17]. There is also a wide range in estimates of COI for dementia in other countries [18]. Of course, there are true differences in the COI of dementia, but the variability in COI figures could also be the result of methodological issues, such as prevalence sources, whether costs of informal care are included and how care is quantified and costed or whether clinical/convenience samples or population-based studies are used as sources for the cost estimates.

The purpose of this paper was to estimate the societal COI of dementia in Sweden in 2012, using different approaches to highlight methodological issues. We also aimed to compare the time trend in COI from 2000 to 2012 when using similar methods. This paper is based on a report that was commissioned by the Swedish National Board of Health and Welfare (NBHW) [19].

## Methods

### COI approach

COI studies can be prevalence- or incidence-based [20]. If the aim is to estimate the economic burden during a certain time period, the prevalence approach is recommended; however, if the aim is to illustrate the economic consequences of policy change (e.g., prevention, treatment, changes in care organisation), the incidence approach may be preferred [21]. Another issue is whether a bottom-up or top-down approach is to be used (or a combination of the two). In a bottom-up study, resource use and costs of a defined (often local) population are described in detail, and, in a step 2, results are extrapolated to a much larger population, such as all persons with the disease in a country. Such results can be based on cross-sectional point estimates or on longitudinal data. Study populations in bottom-up studies can be population-based or be derived from other kinds of study populations (e.g., clinic/hospital-based, convenience samples). In top-down studies, the share of total resource use and cost of care in the region that is attributable to the disease of interest are calculated, often using data from registers and other databases. A combination of the two approaches may also be required, depending on data availability. The distinction between costs for patients with a certain disease (‘gross costs’) and costs due to the disease (‘net costs’) is important; attributability of costs to a specific condition cannot be observed directly, but can only be inferred (e.g., by comparing with costs for subjects without the condition).

The perspective of a COI study defines the viewpoint of the calculations. The societal perspective includes all costs, regardless of the payer, and is recommended by most published guidelines for economic evaluations [22]. In the case of dementia, this means that informal care is assigned a cost. The viewpoint can also be narrowed down, such as a county council, a municipality, an insurance company or a health maintenance organisation.

Costs are often divided into direct costs (‘costs of resources used’ for medical and social/non-medical care) and indirect costs (‘costs of resources lost’ due to production losses because of morbidity and mortality). Informal care by non-professionals such as next of kin may be difficult to classify in these terms. If the informal carers are being remunerated to some extent, it may be regarded as a direct cost, though this often applies to only a low proportion of the total caregiving time. If the carer has partly given up work to care for the PWD, this constitutes an indirect cost equal to the value of the lost productivity. Informal care by retired persons or care during ‘non-working’ time is more complicated to evaluate and assign an opportunity cost, because there is no market for this resource.

In this paper, we apply several methodological approaches. We use a prevalence-based societal perspective in which both bottom-up and top-down methods are employed, and we also present estimates of both gross and net costs.

### Epidemiology

In a prevalence-based COI study, the number of persons with the disease in question is essential. Because individual diagnoses of all PWD are not available, estimates of the number of PWD are necessary. Such estimates are based on demographic statistics (from Statistics Sweden in this paper) and age-specific dementia prevalence figures (e.g., in 5-year classes). In this paper, we use such meta-analysis-based figures presented by the Swedish Agency for Health Technology Assessment (SBU) [23]: 1% for persons aged 60–64 years, 1.5% for 65–69 years, 3% for 70–74 years, 6% for 75–79 years, 13% for 80–84 years, 24% for 85–89 years, 34% for 90–94 years and 45% for 95 years and older. Because there is a discussion currently regarding age-specific dementia prevalence [10], we also present results based on other prevalence sources [3, 8, 9, 24–28] in the sensitivity analysis. In the base option, we assume that in 2012 there were 158,000 PWD in Sweden. The number of incident cases of dementia in Sweden in 2012 (based on demographic statistics and adjusted for prevalent dementia cases) and the meta-analysis by Fratiglioni et al. [29] is estimated at 25,000 persons.

### Care organisation in Sweden

PWD in Sweden receive care using several care alternatives. Basically, health care is provided by doctors, registered and enrolled nurses, occupational therapists and physiotherapists, in the own homes of the elderly, in an institution or in hospitals. The municipality’s social services offer home help, such as help with daily activities (shopping, cooking, cleaning and laundry and/or personal care such as help with feeding, bathing, toileting, getting [un]dressed and into/out of bed). There is also a range of other services, such as home nursing, transportation services, day care, short-term institutional care, meals on wheels, security alarms, housing adaptations and technical aids. A varying amount of informal care is also common at home. (There is also a small amount of informal care in institutions [30].)

Medical direct costs include costs of hospital care, physician clinic visits, physician visits in primary care, visits to emergency units without hospitalisation, visits to and by rehabilitation staff, drug costs and costs of diagnoses. Non-medical direct costs include costs of institutional care, day care and social services. In this paper, indirect costs are restricted to production losses for patients.

To distribute the Swedish population of PWD (158,000 persons) in the care system for the elderly in 2012, we use a combination of results from bottom-up figures, mainly from population-based projects such as the Kungsholmen project [30, 31] and the Swedish National Study on Aging and Care (SNAC) [32, 33], as well as national top-down data [34–38]. Institutional care is divided into three types: nursing homes, group living for PWD and ‘other’ types of sheltered housing (e.g., different types of residential care facilities) [16]. On the basis of population-based studies, 58% of PWD were assumed to live at home and 42% in different kinds of institutional care at various proportions (40–100%) (Table 1).

**Table 1:** Estimated distribution of the Swedish population with dementia in the care system in 2012

	%	Proportion of PWD (%)	Number of PWD
At home	58		91,900
Of those with respite care			3900
Institution	42		66,100
All institutional care			
Higher staffed		75	19,000
Group living		100	30,000
Lower staffed		40	14,000
Institutionalised <65 years old			3100
All			158,000

*PWD* Persons with dementia

### Resource use and costs

The list of potential resources that PWD might use is large, and it is necessary to focus on resource items that are significant cost drivers. All costs are expressed as 2012 SEK (1 € = SEK 8.77, 1 US$ = SEK 6.96).

#### Direct costs

##### Social care sector

Care for PWD in the social care sector consists of support at home by home help, day care, respite care and long-term institutional care.

##### Home care

About 92,000 PWD are estimated to be living at home. For the quantification of support at home, we used the approach of the Resource Utilization in Dementia (RUD) instrument [39], where formal (and informal) care is divided into three proportions: support in personal/basic activities of daily living (PADL), instrumental activities of daily living (IADL) and supervision. For home help services, we used figures based on PADL and IADL. On the basis of Swedish studies where the RUD instrument has been used [40, 41], we estimated the average daily support at 0.5 h/day. This average figure also includes zero users. The hourly cost for formal home care is derived from the Swedish Association of Local Authorities and Regions (SALAR) [42].

##### Day care

The Swedish Dementia Registry (SveDem) was established in 2007. It is an Internet-based quality register where several indicators can be followed over time according to national guidelines, such as diagnostic workup, medical treatment and support from community [43]. On the basis of extrapolations from the annual reports from SveDem [44] (adjusted for incomplete coverage), we estimate that 10,000 PWD have access to day care every year. The annual unit cost for day care is derived from statistics from the NBHW [45].

##### Institutional care

The method of classifying institutional care in Sweden has changed over the years. In a report for the NBHW regarding staffing of long-term institutional care, three levels were used: living in facilities purposely designed for people with dementia (group living, collective living, group dwelling) [36], high-staffed (e.g., nursing homes) and low-staffed (residential care facilities). Table 1 displays the distribution and proportions of PWD in the different forms of institutional care, based on updates of the previous COI estimates [16, 17] and calculations in the Swedish national dementia guidelines [46]. There are also small numbers of PWD younger than 65 years of age as well as PWD using short-term respite care embedded in institutional facilities (annualised rate).

##### Dementia nurse

An expanding and valuable resource in dementia care is the dementia nurse, who organises support for families, coordinates care and also works with overall planning of dementia care. It is estimated that there were about 500 such nurses in 2012 [47]. As unit cost for the dementia nurses, we used the average monthly salary for specially trained nurses, including the social security charges [48].

##### Medical care sector costs

Care for PWD in the medical care sector consists of hospital care, visits to primary care, visits to various specialist clinics (including some of the costs for diagnostic procedures), visits to emergency rooms, use of rehabilitation resources and drug use. The unit costs for these resources are derived mainly from the costing database from SALAR [49].

##### Hospital care

Figures regarding the use of hospital inpatient care are derived from the Swedish National Patient Register for the diagnoses F000, F001, F002, F009, F012, F020, F021, F022, F023, F028, G300, G301, G308 and G309 [50].

##### Outpatient care visits

Similarly to the figures for inpatient hospital care, visits to specialists were derived from the National Patient Register [50]. Regarding other outpatient care visits (e.g., primary care, occupational therapists, physiotherapists), there is no national statistical source available. However, on the basis of data from the SNAC project [51–53], it was estimated that PWD made two visits per year to family physicians and four visits each per year to district nurses, occupational therapists and physiotherapists.

##### Diagnosis

On the basis of an inquiry by the Swedish Ministry of Health to specialist clinics, it was estimated that there were 14,000 diagnostic workups per year in 2003 [34]. In the SveDem register, about 4000 newly diagnosed PWD are registered every year [44]. According to a recent questionnaire sent from SveDem to the memory clinics in Sweden, about 50% of those who went through the diagnostic workup got a final dementia diagnosis. Because the register does not have a completely nationwide coverage, we assume that there still are 14,000 diagnostic workups in specialist care. The figures for primary health care are more difficult to estimate. In SveDem, about 3500 newly diagnosed PWD are registered in primary care. About two-thirds of the primary care centres are part of SveDem, and, given the assumption that 50% also get a dementia diagnosis in primary care, we assume that there are about 10,000 diagnostic procedures in primary care every year. In a paper from SveDem, the costs for diagnostic workup were estimated to be SEK 7017 in primary care and SEK 12,095 on the specialist level. These cost figures are in line with those reported in another Swedish paper from the Kalmar region of Sweden [54].

##### Drug use

The Nordanstig and Kungsholmen SNAC sites in Stockholm include individual and diagnosis-related (e.g., dementia) information about drug use and its related costs [55].

##### Informal care

Quantification and costing of informal care and unpaid work are controversial and complicated issues [56–58]. It is challenging to delimit time spent by caregivers on different caregiving tasks (e.g., supervision) and to determine whether each activity has an opportunity cost (i.e., what is the value of the caregiver’s time in its best alternative use). We relied on several previous studies in which researchers used the RUD framework to estimate caregiving time [39]. To avoid overestimates of informal care, we aimed to use population-based data wherever possible. One such source is the Nordanstig project [40], and now we can also add data regarding the amount of informal care from the database in the SNAC project (baseline 2001–2003 and follow-up 2007–2009). On the basis of information from these sources, we estimate that PWD receive a daily support of 1.9 h in PADL and IADL. We also have data from other Swedish studies on informal care with the RUD instrument [41, 59, 60]. In these studies, the range was 1.5–3.3 h per day in PADL and IADL support. These figures, as well as data on supervision needs, are used in the sensitivity analysis.

Several costing approaches have been discussed [56, 61–64]. In theory, informal care should be valued by the opportunity cost of the carers’ time. However, identifying this opportunity cost is not straightforward [65]. Market prices are not available, except for carers of working age, and, in many cases, it is not apparent what the ‘best alternative use’ of the carers’ time is. The main providers of informal care are spouses and the children/children’s spouses of the PWD. Thus, in the base case, the cost of informal care is weighted and based on studies where the proportion of spouses (two-thirds) and children (one-third) as carers is estimated [33, 40, 66]. For the daughters/daughters in law and sons/sons in law, the average cost per hour of paid work (SEK 270) was used [67], whereas for leisure time, we used the opportunity cost per hour as 35% of the average wage [68]. The resulting weighted hourly cost of informal care was SEK 152. In the sensitivity analysis, other costing approaches are presented.

#### Indirect costs

Most people with dementia are retired, but those younger than age 65 years are of working age. On the basis of register data from the Swedish Social Insurance Agency, 431 persons were prematurely retired or on sick leave due to dementia diagnoses. This is probably a low estimate because PWD often are on sick leave for other diagnoses. The figure is also based on the main diagnosis in the register. Furthermore, early retirement or sick leave by other family members is not incorporated into these figures. The unit cost is based on the average wage for the people aged 55–65 years [67] and not on the transfer costs for the retirement or sick leave. The unit costs are summarised in Table 2. Most of the cost sources were derived from official authorities and registers.

**Table 2:** Unit costs in base case

	Unit cost (SEK)^a^	Reference
Medical care sector		
Hospital care	4780/day	[49]
Emergency room visits	2988/visit	[49]
Outpatient care visits (specialists)	3918/visit	[49]
Primary care physician visits	1140/visit	[83]
Other outpatient care visits	931/visit	[49]
Drug use	7080/year	[55]
Diagnosis in primary care	7017/diagnosis	[84]
Diagnosis in specialist care	12,095/diagnosis	[84]
Social care sector		
Higher-staffed institutional care	606,134/year	[42]
Group living	606,134/year	[42]
Lower-staffed institutional care	472,828/year	[42]
Day care	98,900/year	[45]
Home services	434/h	[42]
Dementia nurse	56,250/year	[48]
Informal care	152/h	[67, 68]
Indirect costs	357,600/year	[67]

^a^1 € corresponds to SEK 8.77 and 1 US$ to SEK 6.96

### Sensitivity analysis

Owing to the variability in underlying sources and different quantification and costing approaches, several other parameters were tested in a sensitivity analysis. There are many other sources for age class prevalence, and in recent years several studies indicating a decline in prevalence have been presented, although some other estimates suggests an increase (European Brain Council [24], Matthews et al. [8], Lobo et al. [25], SNAC-Kungsholmen [9], EURODEM [26], Alzheimer Cooperative Valuation in Europe [27], EuroCoDe [28] and Alzheimer’s Disease International [ADI] [3]). Owing to the discussions regarding the quantification and costing of informal care, several alternative options have been tested: the amount of informal care with supervision added to activities of daily living (in total 4.7 h/day), alternative sources for the amount of informal care (1.5–5.3 h/day in PADL and IADL) [41, 59, 60, 69], various costing of informal care (a zero value, a replacement cost instead of opportunity cost, SEK 434/h [42] and an alternative source for the opportunity cost [SEK 31/h, based on the Swedish Road Association] [70]). Institutional care costs are one of the heaviest cost drivers in dementia care. Figures regarding the proportion of PWD in different forms of institutional care also vary [30, 32, 33, 51–53, 71], and thus we test an option where the proportion of PWD is lower. The unit costs for institutional care are also varied (25th and 75th percentiles).

### Cost attributable to dementia (‘net’ costs)

The costs presented as the main option can be regarded as ‘gross costs’ because they comprise the costs of PWD including costs for conditions other than dementia. One way to estimate costs that are attributable to dementia (‘net costs’) is to calculate the cost difference between costs for PWD and for people without dementia. The estimates are based on the total direct costs of care for the elderly in Sweden (65 years old and above), which in 2012 were SEK 182 billion, covering 102 billion SEK in the social care sector (municipalities) and SEK 80 billion in the health care sector (the county councils) [38]. Estimates are possible for the population 65 years and older, which represented about 148,000 of the 158,000 PWD in 2012. It is assumed that the costs for PWD aged 65 years and older are the same as those for all PWD. The influence of production losses on PWD was so low that this was not included in the ‘net’ cost estimates. The net costs can be estimated using a step-by-step procedure (see Table 7 in the Results section below).

## Results

The total societal costs of dementia in Sweden in 2012 were in the base case estimated at about SEK 63 billion (Table 3). By far the most important cost driver is the cost of long-term institutional care (SEK 38 billion). Furthermore, almost 80% of the total costs occur in the municipalities’ social care sector. The cost of informal care constitutes 17%, whilst the medical care sector within the county council represents only a small proportion (5%). The costs of drug use constitute almost 39% of the health care sector costs.

**Table 3:** Total societal costs of dementia in Sweden in 2012 (base option)

	Cost (million SEK)^a^	Per PWD, SEK^a^	Proportion
Medical care sector (county councils)			
Hospital care	276		0.4%
Emergency room visits	264		0.4%
Outpatient care visits (specialists)	57		0.1%
Primary care physician visits	360		0.6%
Other outpatient care (e.g., rehabilitation)	589		0.9%
Drug use	1120		1.8%
Diagnostic workups	240		0.4%
Total medical care sector	2904	18,382	4.6%
Social care sector (municipalities)			
Institutional care (permanent)	38,199		60.7%
Short-term respite care	2364		3.8%
Day care	989		1.6%
Home services	7372		11.7%
Dementia nurses	338		0.5%
Total social care sector	49,262	311,783	78.3%
Indirect costs	118	744	0.2%
Total^b^	62,920	398,226	100.0%

*PWD* Persons with dementia, *SEK* Swedish krona

^a^1 € corresponds to SEK 8.77 and 1 US$ to SEK 6.96.

^b^Discrepancies are due to rounding

When comparing with the previous COI studies [16, 17], some adjustments are needed because the 2012 figures comprise more cost items. The total costs are rather similar in 2000, 2005 and 2012 (Table 4), but while the number of PWD has increased, the cost per PWD has decreased. The main reason for the reduced cost per PWD is that the proportion of PWD who are estimated to be living in institutions has decreased from about 56% in 2000 to 42% in 2012; during that period, the number of institutional beds in Sweden was reduced from about 120,000 in 2000 to 95,000 in 2012.

**Table 4:** Societal cost of dementia (in millions SEK, adjusted to cost level of 2012) in 2012, 2005 and 2000 in different sectors

	2012		2005		2000	
	Cost^a^	%	Cost^a^	%	Cost	%
Medical care sector	2848	5.0%	2701	5.0%	3277	5.9%
Social care sector	44,681	78.1%	43,275	79.4%	45,542	82.5%
Informal care	9534	16.7%	8065	14.8%	6017	10.9%
Indirect costs	118	0.2%	447	0.8%	358	0.6%
Total costs	57,181	100.0%	54,487	100.0%	55,194	100.0%
Number of PWD	158,000		142,200		133,000	
Cost per PWD (SEK)	361,902		383,173		414,989	

*PWD* Persons with dementia, *SEK* Swedish krona

^a^1 € corresponds to SEK 8.77 and 1 US$ to SEK 6.96. Cost items are adjusted to be similar; thus, costs are not identical to Table 3 for 2012

The most striking finding in the sensitivity analysis is the great range in costs with different approaches of costing informal care (range SEK 7.6 billion–71.5 billion) with a corresponding great variation in total societal cost, ranging from SEK 52 billion to SEK 124 billion (Table 5).

**Table 5:** One-way sensitivity analysis of costs of informal care

	Hours/day	Hourly cost	Total cost^a^ (billion SEK)	Informal care^a^ (billion SEK)	Cost per PWD^a^ (SEK)
Base case	1.9	152	62.9	10.6	398,000
Higher amount of hours	3.3	152	70.2	18.0	445,000
Supervision time added	4.7	152	77.4	25.4	490,000
Replacement cost	1.9	434	82.6	30.3	523,000
Supervision time added and replacement cost	4.7	434	123.8	71.5	784,000
Alternative opportunity cost	1.9	109	59.9	7.6	379,000
Zero cost for informal care	0	0	52.3	0	331,000

*PWD* Persons with dementia, *SEK* Swedish krona

^a^1 € corresponds to SEK 8.77 and 1 US$ to SEK 6.96

In the other options in the sensitivity analysis (Table 6), the differences vs the base case were smaller. The rather low variation of costs with different prevalence sources is due to an assumed similar proportion of PWD in institutions.

**Table 6:** Other one-way sensitivity analyses

Varied entity	Value	Cost^a^ (billion SEK)	Cost per PWD	Source [reference]
Prevalence (number of PWD)	158,000	62.9	398,000	Base case
Prevalence	113,000	54.1	478,000	EBC [24]
Prevalence	117,000	54.8	469,000	Matthews et al. [8]
Prevalence	123,000	56.0	455,000	Lobo et al. [25]
Prevalence	128,000	56.6	453,000	SNAC-K [9]
Prevalence	148,000	60.9	412,000	EURODEM [26]
Prevalence	151,000	61.5	408,000	ALCOVE [27]
Prevalence	168,000	64.9	386,000	EuroCoDe [28]
Prevalence	169,000	65.1	385,000	ADI [3]
Home care: per executed hour	558	63.8	404,000	
Home care: median hourly cost	420	61.6	390,000	[42]
Home care: 25th percentile per hour	378	60.9	385,000	[42]
Home care: 75th percentile per hour	473	62.5	395,000	[42]
Institutional care: 25th percentile (low staffed and high staffed)	363,920 and 565,811	59.1	374,000	[42]
Institutional care: 75th percentile (low staffed and high staffed)	534,142 and 639,828	65.6	416,000	
Institutional care: proportion PWD	50% in low-staffed, 80% in high-staffed	64.2	406,000	
Institutional care: proportion PWD	40% in low-staffed, 50% in high-staffed	59.1	374,000	

*PWD* Persons with dementia, *SEK* Swedish krona

^a^1 € corresponds to SEK 8.77 and 1 US$ to SEK 6.96

*EBC* European Brain Council

The direct cost that we assumed was related to dementia solely (‘net costs’) was about SEK 40 billion (Table 7), which constitutes about 76% of the ‘gross’ direct costs (and about 80% of total societal costs if it is assumed that all informal care is related to dementia).

**Table 7:** Estimated ‘net’ costs of dementia (SEK)

	Social care sector	Medical care sector	Total
Step 1			
Direct cost of care for people aged 65+ years	101,756	79,973	181,729 million SEK
Direct costs of dementia	49,262	2904	52,166 million SEK
Direct costs for PWD aged 65+ years^a^	46,144	2720	48,864 million SEK
Direct costs of care for people without dementia aged 65+ years			132,865 million SEK
Number of people aged 65+ years			1,828,283
Number of people aged 65+ years without dementia			1,680,283
Direct cost per person aged 65+ years without dementia			79,073 SEK
Step 2: costs per PWD			
Gross cost per person with dementia^a^ (Table 2)^b^			398,226 SEK
Exclude: direct cost for person without dementia as above			79,073 SEK
Exclude: costs of informal care of PWD (Table 2)			67,318 SEK
‘Net’ direct costs			251,836 SEK
‘Net’ total costs (including informal care)			319,154 SEK
Step 3: aggregated costs			
Aggregated ‘net’ direct costs of dementia aged 65+ years (148,000 PWD)			37,272 million SEK
Aggregated ‘net’ direct costs all dementia (158,000 PWD)			39,790 million SEK
Aggregated ‘net’ costs all including informal care aged 65+ years (148,000 PWD)			47,235 million SEK
Aggregated ‘net’ costs all including informal care all dementia (158,000 PWD)			50,426 million SEK

*PWD* Persons with dementia, *SEK* Swedish krona

^a^Assumptions: similar for PWD aged 65+ years as for all PWD and adjustment factor (here, dementia aged 65+ years only) 148,000/158,000

^b^1 € corresponds to SEK 8.77 and 1 US$ to SEK 6.96

## Discussion

Three major findings are obvious: (1) The costs of dementia care are very extensive; (2) the majority of the costs occur in the social care sector (the municipalities); and (3) the quantification and costing of informal care are crucial for the cost estimates. When the Swedish figures (total costs of SEK 400,000 [approximately € 45,000, approximately US$ 57,000]) are compared with other COI figures, the need for transparency is clear. In a review, Jönsson and Wimo found a range between €6000 and €64,000 per case in different European countries, where the main reason for this variation was methodological issues [72]. A similar variation in worldwide COI studies was found in the dementia report by the SBU [23]. In the worldwide cost estimates presented by ADI [1], it was concluded that, besides methodological issues, the interaction between informal care and the social care sector (including, e.g. long-term institutional care) was crucial for the cost differences: low/no resources for long-term institutional care resulted in a high proportion of the costs of informal care and vice versa.

The most important cost driver in Sweden is the cost of institutional care. The institutional concept is wide and includes a variety of care settings where the least common denominator, in our opinion, is staffing around the clock. We have used three institutional levels (nursing homes, dementia-designated care such as group living [73] and residential care alternatives for the aged or similar) to cover the different content of care and costs, and in the sensitivity analysis we have a further variation in costs of institutional care. Although some range occurs in the costs of institutional care in the sensitivity analysis, it is not of the same magnitude as when different approaches for the quantification and costing of informal care were tested [61]. In the literature, there is an extensive variation in the quantity, ranging from 1.5 h to 16 h per day [61, 72, 74–76]. This great variation depends mainly on how many components of informal care are included; the lower figures represent only PADL support, while the higher figures also include IADLs and supervision/surveillance. Another factor is whether the sources of informal care are based on clinical/convenience samples or on population-based studies, the latter including zero values with lower hourly figures as a result. The costing issue is also crucial, and several methods have been proposed [56, 61–64]. The assumptions used for the mix between informal carers of working age and retired carers also have implications for the costing approach as well as for long-term care policies [77]. Although the opportunity cost is recommended by most economists, it is not always easy to identify it [65]. Market prices are not available, except for carers of working age, and, in many cases, it is not apparent what the ‘best alternative use’ of the carers’ time is. Thus replacement costs or average wage or suchlike are used in many COI studies, most often resulting in higher costs. Because the different methods of quantification and costing of informal care resulted in such a great difference in final costs, it is obvious that this issue needs transparency but must also be a target for future methodological discussions. Validated instruments are essential where it is clear how informal care is quantified as well as a transparent presentation of how unit costs for informal care are obtained and valued. A sensitivity analysis is crucial to highlight the variability and to make comparisons with other studies possible.

The relatively high proportion (39%) of drug costs in the total costs of the county councils may have several explanations. First, the county councils pay for all drug use of PWD, even if, for example, they are cared for in municipal nursing homes. Second, the costs of hospital care as well as the costs of outpatient specialist care are based on register data, where a diagnosis of dementia often is not registered even if a patient has a dementia disorder [78]. Thus, these costs may be underestimated, resulting in a relatively higher proportion of the costs of drugs for the county councils. In contrast, even if there is a debate regarding the prevalence of dementia [7–11], the use of different prevalence sources in the sensitivity analysis did not alter the COI figures significantly.

Although the societal costs of dementia are substantial, the costs per person were lower in 2012 than in 2000 and 2005. The main reason is the de-institutionalisation trend in Sweden. The number of long-term care beds in institutions in Sweden (persons aged 65 years and above) decreased from about 119,000 in 2000 to 100,400 in 2005 and 90,500 in 2012 [79], which resulted in lower costs (per person) in 2012 than in 2000 and 2005. Consequently, the relative impact of costs of informal care has increased. This reflects, at the same time, a gradual shift of responsibilities from the state to the families. The question whether this is good or bad cannot be answered by a COI study. There are no assessments of the quality of care or the quality of life of PWD or the informal carers in our data. Such consequences can be analysed with instruments such as DEMQOL (for PWD) [80] or CarerQol (for informal carers) [81], and they are usually incorporated as outcomes in cost-effectiveness analysis of interventions. In light of expected demographic changes, with a considerably rapid increase in the numbers of people aged 80 years and older after 2020 [82], these trends in the change of the care structure need a profound analysis.

It is obvious that transparency is crucial in COI studies, otherwise comparisons are not meaningful. A comprehensive and transparent sensitivity analysis also highlights different analytical approaches, which makes comparisons with other COI studies easier.

## Conclusions

The societal costs of dementia are very high. The cost per PWD has decreased somewhat, mainly because of de-institutionalisation. The majority of the costs occur in the social care sector, but the costing of informal care is crucial for the cost estimates.

## Abbreviations

ADI: Alzheimer’s Disease International
ALCOVE: Alzheimer Cooperative Valuation in Europe
EBC: European Brain Council
COI: Cost of illness
IADL: Instrumental activities of daily living
NBHW: Swedish National Board of Health and Welfare
PADL: Personal activities of daily living
PWD: Persons with dementia
RUD: Resource Utilization in Dementia
SALAR: Swedish Association of Local Authorities and Regions
SBU: Swedish Agency for Health Technology Assessment
SEK: Swedish krona
SNAC: Swedish National Study on Aging and Care
SveDem: Swedish Dementia Registry

## References

[1] Wimo A, Jönsson L, Bond J, Prince M, Winblad B (2013). Alzheimer Disease International. The worldwide economic impact of dementia 2010. Alzheimers Dement.

[2] Wimo A, Prince M (2010). World Alzheimer Report 2010: the global economic impact of dementia.

[3] Prince M, Bryce R, Albanese E, Wimo A, Ribeiro W, Ferri CP (2013). The global prevalence of dementia: a systematic review and metaanalysis. Alzheimers Dement.

[4] Wimo A, Winblad B, Aguero-Torres H, von Strauss E (2003). The magnitude of dementia occurrence in the world. Alzheimer Dis Assoc Disord.

[5] Ferri CP, Prince M, Brayne C, Brodaty H, Fratiglioni L, Ganguli M (2005). Global prevalence of dementia: a Delphi consensus study. Lancet.

[6] Lovestone S (2002). Can we afford to develop treatments for dementia?. J Neurol Neurosurg Psychiatry.

[7] Schrijvers EM, Verhaaren BF, Koudstaal PJ, Hofman A, Ikram MA, Breteler MM (2012). Is dementia incidence declining? Trends in dementia incidence since 1990 in the Rotterdam Study. Neurology.

[8] Matthews FE, Arthur A, Barnes LE, Bond J, Jagger C, Robinson L (2013). A two-decade comparison of prevalence of dementia in individuals aged 65 years and older from three geographical areas of England: results of the Cognitive Function and Ageing Study I and II. Lancet..

[9] Qiu C, von Strauss E, Bäckman L, Winblad B, Fratiglioni L (2013). Twenty-year changes in dementia occurrence suggest decreasing incidence in central Stockholm. Sweden. Neurology..

[10] Rocca WA, Petersen RC, Knopman DS, Hebert LE, Evans DA, Hall KS (2011). Trends in the incidence and prevalence of Alzheimer’s disease, dementia, and cognitive impairment in the United States. Alzheimers Dement.

[11] Langa KM, Larson EB, Karlawish JH, Cutler DM, Kabeto MU, Kim SY (2008). Trends in the prevalence and mortality of cognitive impairment in the United States: is there evidence of a compression of cognitive morbidity?. Alzheimers Dement.

[12] Schneider LS, Mangialasche F, Andreasen N, Feldman H, Giacobini E, Jones R (2014). Clinical trials and late-stage drug development for Alzheimer’s disease: an appraisal from 1984 to 2014. J Intern Med.

[13] Mangialasche F, Solomon A, Winblad B, Mecocci P, Kivipelto M (2010). Alzheimer’s disease: clinical trials and drug development. Lancet Neurol.

[14] OECD Health Statistics [database on the Internet]. OECD. 2014. http://stats.oecd.org/index.aspx?DataSetCode=HEALTH_STAT. Accessed 14 Dec 2014.

[15] Wimo A, Karlsson G, Sandman PO, Corder L, Winblad B (1997). Cost of illness due to dementia in Sweden. Int J Geriatr Psychiatry.

[16] Wimo A, Jönsson L (2011). The societal costs of dementia [in Swedish].

[17] Wimo A, Johansson L, Jönsson L (2007). The societal costs of dementia and the number of demented in Sweden 2005 [in Swedish]. Report 2007-123-32.

[18] Swedish Agency for Health Technology Assessment (SBU) (2008). Dementia: a systematic review. Report 172E/1-3.

[19] Wimo A, Jönsson L, Fratiglioni L, Sandman P, Gustavsson A, Sköldunger A (2011). Demenssjukdomarnas samhällskostnader i Sverige 2012. Report 2014-6-3.

[20] Rice DP, Hodgson TA, Kopstein AN (1985). The economic costs of illness: a replication and update. Health Care Financ Rev.

[21] Andlin-Sobocki P, Jönsson B, Wittchen HU, Olesen J (2005). Cost of disorders of the brain in Europe. Eur J Neurol..

[22] Siegel JE, Torrance GW, Russell LB, Luce BR, Weinstein MC (1997). Gold MR; Panel on Cost Effectiveness in Health and Medicine. Guidelines for pharmacoeconomic studies: recommendations from the panel on cost effectiveness in health and medicine. Pharmacoeconomics.

[23] Swedish Agency for Technology Assessment in Health Care (SBU) (2006). Dementia [in Swedish].

[24] Gustavsson A, Svensson M, Jacobi F, Allgulander C, Alonso J, Beghi E (2013). Cost of disorders of the brain in Europe 2010. Eur Neuropsychopharmacol.

[25] Lobo A, Launer LJ, Fratiglioni L, Andersen K, Di Carlo A, Breteler MM (2000). Neurologic Diseases in the Elderly Research Group. Prevalence of dementia and major subtypes in Europe: a collaborative study of population-based cohorts. Neurology.

[26] Hofman A, Rocca WA, Brayne C, Breteler MM, Clarke M, Cooper B (1991). EURODEM Prevalence Research Group. The prevalence of dementia in Europe: a collaborative study of 1980–1990 findings. Int J Epidemiol.

[27] Alzheimer Cooperative Valuation in Europe (ALCOVE) (2013). ALCOVE synthetic report 2013: a general presentation of ALCOVE.

[28] European Collaboration on Dementia (EuroCoDe) (2009). WP7 - prevalence rates.

[29] Fratiglioni L, Launer LJ, Andersen K, Breteler MM, Copeland JR, Dartigues JF (2000). Neurologic Diseases in the Elderly Research Group. Incidence of dementia and major subtypes in Europe: a collaborative study of population-based cohorts. Neurology.

[30] Nordberg G, Wimo A, Jönsson L, Kåreholt I, Sjölund B, Lagergren M (2007). Time use and costs of institutionalised elderly persons with or without dementia: results from the Nordanstig cohort in the Kungsholmen Project - a population based study in Sweden. Int J Geriatric Psychiatry.

[31] Fratiglioni L, Viitanen M, Backman L, Sandman PO, Winblad B (1992). Occurrence of dementia in advanced age: the study design of the Kungsholmen Project. Neuroepidemiology..

[32] Lagergren M, Fratiglioni L, Hallberg IR, Berglund J, Elmståhl S, Hagberg B (2004). A longitudinal study integrating population, care and social services data: the Swedish National study on Aging and Care (SNAC). Aging Clin Exp Res.

[33] Lagergren M, Holst G, Rahm-Hallberg I, Wimo A (2002). Behov och insatser för de äldre i SNAC-kommunerna. Jämförelser från SNAC baslinjeundersökningar 2001 i Karlskrona, på Kungsholmen, i Nordanstig och i Skåne. SNAC-K rapport 3.

[34] Socialdepartementet (2003). På väg mot en god demensvård: samhällets insatser för personer med demenssjukdomar och deras anhöriga.

[35] Socialstyrelsen (2007). Vård och omsorg om äldre. Report 2007-131-12.

[36] Socialstyrelsen (2012). Konsekvensutredning – förslag till föreskrifter och allmänna råd om ansvaret för personer med demenssjukdom och bemanning i sär-skilda boenden. Report Dnr 7993/2011.

[37] Socialstyrelsen (2014). Nationell utvärdering – vård och omsorg vid demenssjukdom 2014: rekommendationer, bedömningar och sammanfattning. Report 2014-2-4.

[38] Socialstyrelsen (2014). Tillståndet och utvecklingen inom hälso - och sjukvård och socialtjänst: lägesrapport 2014. Report 2014-2-3.

[39] Wimo A, Wetterholm AL, Mastey V, Winblad B, Wimo A, Jönsson B, Karlsson G, Winblad B (1998). Evaluation of the healthcare resource utilization and caregiver time in anti-dementia drug trials. Health economics of dementia.

[40] Nordberg G, von Strauss E, Kåreholt I, Johansson L, Wimo A (2005). The amount of informal and formal care among non-demented and demented elderly persons—results from a Swedish population-based study. Int J Geriatr Psychiatry.

[41] Mesterton J, Wimo A, By A, Langworth S, Winblad B, Jönsson L (2010). Cross sectional observational study on the societal costs of Alzheimer’s disease. Curr Alzheimer Res..

[42] Sveriges Kommuner och Landsting (SKL) (2012). Kostnad per brukare - Jämförelser mellan kommuner inom omsorg om äldre och personer med funktionsnedsättning – utfall 2011.

[43] Religa D, Spangberg K, Wimo A, Edlund AK, Winblad B, Eriksdotter-Jonhagen M (2012). Dementia diagnosis differs in men and women and depends on age and dementia severity: data from SveDem, the Swedish Dementia Quality Registry. Dement Geriatr Cogn Disord.

[44] SveDem (2013). Svenska Demensregistret: årsrapport 2012.

[45] Socialstyrelsen (2013). Kostnadsmått 2012: öppna jämförelser av socialtjänsten 2013. Report 2013-10-1.

[46] Socialstyrelsen (2010). Nationella riktlinjer för vård och omsorg vid demenssjukdom 2010: stöd för styrning och ledning.

[47] Dahlrup B. Nationellt nätverk för Demenssjuksköterskor. Malmö, Sweden: Nationellt nätverk för Demenssjuksköterskor; 2013. http://www.swenurse.se/Sektioner-och-Natverk/Nationellt-natverk-for-demenssjukskoterskor/Om-oss/ (in Swedish). Accessed 31 Oct 2016.

[48] Statistics Sweden (SCB). Genomsnittlig månadslön inom primärkommunal sektor efter yrke, ålder, kön och år: 2012. http://www.statistikdatabasen.scb.se/pxweb/sv/ssd/?rxid=6fef5754-338d-467c-bb2b-3c393b95d7bf (in Swedish). Accessed 22 Aug 2013.

[49] Kostnad per patient (KPP) database. Sveriges Kommuner och Landsting (SKL). 2013. https://skl.se/ekonomijuridikstatistik/statistik/kostnadperpatientkpp.1076.html (in Swedish). Accessed 22 Aug 2013.

[50] Socialstyrelsens statistikdatabas, 2013. http://www.socialstyrelsen.se/statistik/statistikdatabas/diagnoserislutenvard (in Swedish). Accessed 22 Aug 2013.

[51] Wimo A, Persson G, Sjölund BM, Lagergren M (2002). SNAC i Nordanstig – Resultat från delprojektet Vårdsystem – baslinjeundersökning våren 2001. Report SNAC-rapport 4.

[52] Osterman J, Lagergren M, Lundberg L (2004). Vårdkonsumtion bland äldre boende på Kungsholmen och Essingeöarna. SNAC-K rapport 6.

[53] Hallberg IR, Karlsson S, Westergren A, Dozet A, Lithman T, Elmstahl S (2001). Kommunal och regional vård till äldre: vård och omsorg i Eslöv, Hässleholm, Malmö, Osby och Ystad till personer 65 år och däröver, våren 2001. Report 1: En delstudie i SNAC (GÅS).

[54] Jedenius E, Wimo A, Strömkvist J, Winblad B, Andreasen N (2007). Dementia program in primary care, five years of experience [abstract]. Int Psychogeriatr..

[55] Sköldunger A (2015). Dementia and use of drugs: economic modelling and population-based studies. Thesis.

[56] van den Berg B, Brouwer WB, Koopmanschap MA (2004). Economic valuation of informal care: an overview of methods and applications. Eur J Health Econ.

[57] van den Berg B, Spauwen P (2006). Measurement of informal care: an empirical study into the valid measurement of time spent on informal caregiving. Health Econ.

[58] Jönsson L (2003). Economic evaluation of treatments for Alzheimer’s disease.

[59] Gustavsson A, Brinck P, Bergvall N, Kolasa K, Wimo A, Winblad B (2011). Predictors of costs of care in Alzheimer’s disease: a multinational sample of 1222 patients. Alzheimers Dement.

[60] Gustavsson A, Jönsson L, Rapp T, Reynish E, Ousset PJ, Andrieu S (2010). Differences in resource use and costs of dementia care between European countries: baseline data from the ICTUS study. J Nutr Health Aging.

[61] McDaid D (2001). Estimating the costs of informal care for people with Alzheimer’s disease: methodological and practical challenges. Int J Geriatr Psychiatry.

[62] Karlsson G, Jönsson B, Wimo A, Winblad B, Wimo A, Jönsson B, Karlsson G, Winblad B (1998). Methodological issues in health economics of dementia. Health economics of dementia.

[63] Lanska DJ (2002). Time is money—or is it? Estimating the costs of informal caregiving. Neurology.

[64] Fukami M (1998). Monetary valuation of unpaid work in 1996—Japan.

[65] Drummond MF, O’Brien B, Stoddart GL, Torrance GW (1997). Methods for the economic evaluation of health care programmes.

[66] Wimo A, von Strauss E, Nordberg G, Sassi F, Johansson L (2002). Time spent on informal and formal care giving for persons with dementia in Sweden. Health Policy.

[67] Genomsnittlig månadslön, kronor efter sektor, yrke, kön och tid, samtliga sektorer, samtliga yrken 2012. Stockholm: Statistiska Centralbyrån. 2013. http://www.statistikdatabasen.scb.se/pxweb/sv/ssd/?rxid=6fef5754-338d-467c-bb2b-3c393b95d7bf (in Swedish). Accessed on 10 Dec 2013.

[68] Johannesson M, Borgquist L, Jönsson B, Rastam L (1991). The costs of treating hypertension—an analysis of different cut-off points. Health Policy.

[69] Wimo A, Elmståhl S, Fratiglioni L, Sjölund BM, Sköldunger A, Fagerström C, Berglund J, Lagergren M. Formal and informal care of community-living older people: A population-based study from the Swedish National study on Aging and Care. J Nutr Health Aging (in press). 10.1007/s12603-016-0747-5 PMC12879159 27999845

[70] Vägverket (1997). Vägverkets samhällsekonomiska kalkylmodell: ekonomisk teori och värderingar.

[71] Lagergren M, Hallberg IR, Holst G, Wimo A (2003). Behov och insatser för de äldre i SNAC-kommunerna.

[72] Jönsson L, Wimo A (2009). The cost of dementia in Europe: a review of the evidence, and methodological considerations. Pharmacoeconomics..

[73] Wimo A, Mattson B, Krakau I, Eriksson T, Nelvig A, Karlsson G (1995). Cost-utility analysis of group living in dementia care. Int J Technol Assess Health Care.

[74] Alzheimer’s Disease International (ADI) (2010). World Alzheimer Report 2010: the global economic impact of dementia.

[75] Hurd MD, Martorell P, Langa KM (2013). Monetary costs of dementia in the United States. N Engl J Med.

[76] Knapp M, Prince M. Dementia UK. London: Alzheimer’s Society; 2007. https://www.alzheimers.org.uk/site/scripts/documents_info.php?documentID=2759. Accessed 31 Oct 2016.

[77] Colombo F, Llena-Nozal A, Mercier J, Tjadens F (2011). Help wanted? Providing and paying for long-term care.

[78] Alzheimer’s Disease International (ADI) (2011). World Alzheimer Report 2011: the benefits of early diagnosis and intervention.

[79] Socialstyrelsen (2013). Äldre och personer med funktionsnedsättning – regiform år 2012: vissa kommunala insatser enligt socialtjänstlagen. Contract 2013-3-23.

[80] Smith SC, Lamping DL, Banerjee S, Harwood RH, Foley B, Smith P (2007). Development of a new measure of health-related quality of life for people with dementia: DEMQOL. Psychol Med.

[81] Brouwer WB, van Exel NJ, van Gorp B, Redekop WK (2006). The CarerQol instrument: a new instrument to measure care-related quality of life of informal caregivers for use in economic evaluations. Qual Life Res.

[82] Statistics Sweden. Population projections. 2014. http://www.statistikdatabasen.scb.se/pxweb/sv/ssd/?rxid=377526ed-23eb-4938-bb38-8a81b07d66a2 (in Swedish). Accessed 14 Dec 2014.

[83] Socialstyrelsen (2005). Patientrelaterad redovisning av verksamhet och kostnader (KPP) inom primärvård: metod, genomförande och användning med exempel från Borlänge sjukhus. Report 2005-124-5.

[84] Wimo A, Religa D, Spangberg K, Edlund AK, Winblad B, Eriksdotter M (2013). Costs of diagnosing dementia: results from SveDem, the Swedish Dementia Registry. Int J Geriatr Psychiatry.

