# Erratum to: The societal costs of dementia in Sweden 2012 – relevance and methodological challenges in valuing informal care

**DOI:** 10.1186/s13195-017-0244-z

**Published:** 2017-02-27

**Authors:** Anders Wimo, Linus Jönsson, Laura Fratiglioni, Per Olof Sandman, Anders Gustavsson, Anders Sköldunger, John Lennarth Johansson

**Affiliations:** 1 Aging Research Centre, Department of Neurobiology, Care Sciences and Society (NVS), Karolinska Institutet and Stockholm University, Stockholm, Sweden; 2 Division of Neurogeriatrics, Department of Neurobiology, Care Sciences and Society (NVS), Karolinska Institutet, Huddinge, Stockholm Sweden; 3 Centre for Research & Development, Uppsala University/County Council of Gävleborg, Gävle, Sweden; 4 Division of Caring Sciences, Department of Neurobiology, Care Sciences and Society (NVS), Karolinska Institutet, Stockholm, Sweden; 5 Department of Nursing, Umeå University, Umeå, Sweden; 6 Department of Health Sciences, Luleå University of Technology, Luleå, Sweden; 7 Stockholm Gerontology Research Centre, Stockholm, Sweden

## Erratum

During production of the original article [1], the line referring to “Informal Care” was omitted from Table 3. It should have been mentioned between “Total social care sector” and “Indirect costs”.

**Table 3:** Total societal costs of dementia in Sweden in 2012 (base option)

	Cost (million SEK)^a^	Per PWD, SEK^a^	Proportion
Medical care sector (county councils)			
Hospital care	276		0.4%
Emergency room visits	264		0.4%
Outpatient care visits (specialists)	57		0.1%
Primary care physician visits	360		0.6%
Other outpatient care (e.g., rehabilitation)	589		0.9%
Drug use	1120		1.8%
Diagnostic work ups	240		0.4%
Total medical care sector	2904	18,382	4.6%
Social care sector (municipalities)			
Institutional care (permanent)	38,199		60.7%
Short-term respite care	2364		3.8%
Day Care	989		1.6%
Home services	7372		11.7%
Dementia nurses	338		0.5%
Total social care sector	49,262	311,783	78.3%
Informal care	10,636	67,318	16.9%
Indirect costs	118	744	0.2%
Total^b^	62,920	398,226	100.0%

*PWD* Persons with dementia, *SEK* Swedish krona

^a^1 € corresponds to SEK 8.77 and 1 US$ to SEK 6.96

^b^Discrepancies due to rounding
